# 5-aminolevulinic acid-mediated photodynamic therapy can target aggressive adult T cell leukemia/lymphoma resistant to conventional chemotherapy

**DOI:** 10.1038/s41598-020-74174-x

**Published:** 2020-10-14

**Authors:** Yasuhisa Sando, Ken-ichi Matsuoka, Yuichi Sumii, Takumi Kondo, Shuntaro Ikegawa, Hiroyuki Sugiura, Makoto Nakamura, Miki Iwamoto, Yusuke Meguri, Noboru Asada, Daisuke Ennishi, Hisakazu Nishimori, Keiko Fujii, Nobuharu Fujii, Atae Utsunomiya, Takashi Oka, Yoshinobu Maeda

**Affiliations:** 1 Department of Hematology and Oncology, Okayama University Graduate School of Medicine, Dentistry and Pharmaceutical Sciences, 2-5-1 Shikata-cho, Kita-ku, Okayama, Okayama 700-8558 Japan; 2 Department of Hematology, Imamura General Hospital, Kagoshima, Japan

**Keywords:** T-cell lymphoma, Targeted therapies

## Abstract

Photodynamic therapy (PDT) is an emerging treatment for various solid cancers. We recently reported that tumor cell lines and patient specimens from adult T cell leukemia/lymphoma (ATL) are susceptible to specific cell death by visible light exposure after a short-term culture with 5-aminolevulinic acid, indicating that extracorporeal photopheresis could eradicate hematological tumor cells circulating in peripheral blood. As a bridge from basic research to clinical trial of PDT for hematological malignancies, we here examined the efficacy of ALA-PDT on various lymphoid malignancies with circulating tumor cells in peripheral blood. We also examined the effects of ALA-PDT on tumor cells before and after conventional chemotherapy. With 16 primary blood samples from 13 patients, we demonstrated that PDT efficiently killed tumor cells without influencing normal lymphocytes in aggressive diseases such as acute ATL. Importantly, PDT could eradicate acute ATL cells remaining after standard chemotherapy or anti-CCR4 antibody, suggesting that PDT could work together with other conventional therapies in a complementary manner. The responses of PDT on indolent tumor cells were various but were clearly depending on accumulation of protoporphyrin IX, which indicates the possibility of biomarker-guided application of PDT. These findings provide important information for developing novel therapeutic strategy for hematological malignancies.

## Introduction

Photodynamic therapy (PDT) is a therapeutic modality that specifically kills target cancer cells through the combination of a photosensitizer and light irradiation. PDT is used for skin diseases such as actinic keratosis because it can be administered repeatedly and does not cause scars^1^. In addition, PDT has also been studied for malignant diseases such as head and neck cancer, esophageal cancer, prostate cancer and bladder cancer^2–7^. A natural amino acid, 5-aminolevulinic acid (5-ALA), is a precursor of protoporphyrin IX (PpIX) in the heme biosynthesis pathway. PpIX is a fluorescent photosensitizer that generates singlet oxygen (^1^O2) in cells exposed to visible light. PpIX selectively accumulates in tumor cells because of metabolic abnormalities. Since tumor cells are abnormal in the heme biosynthetic pathway, PpIX specifically accumulates in tumor cells. By this property, 5-ALA is used for photodynamic diagnosis (PDD) and PDT^8,9^. For malignant glioma and bladder cancer, intraoperative visualization with 5-ALA can remove tumors more completely than without 5-ALA^10,11^. ALA-PDT has also been approved to treat the precancerous disease actinic keratosis^12^. However, there are only a few studies on the application of ALA-PDT for hematological malignancies^13,14^.

Adult T cell leukemia/lymphoma (ATL) is an aggressive T cell malignancy caused by human T cell leukemia virus type 1 (HTLV-1)^15^. ATL is classified into 4 clinical subtypes: acute, lymphoma, chronic, and smoldering^16^. The acute and lymphoma types of ATL, which are considered aggressive ATL, have a dismal prognosis, mainly because of resistance to a variety of cytotoxic agents^17–19^. For aggressive ATL patients, allogeneic hematopoietic stem cell transplantation (HSCT) has a critical role in maintaining long-term remission^20–23^. As a bridge to HSCT, patients need to receive intensive combination chemotherapy to reduce the tumor burden, however, many cases could become refractory to chemotherapy before transplant. In recent years, the efficacy of humanized anti-chemokine receptor 4 (CCR4) antibodies and immunomodulatory drugs such as lenalidomide have been approved and used in the treatment of aggressive ATL^24–27^. However, the pretransplant use of anti-CCR4 antibodies or lenalidomide could cause severe graft-versus-host disease (GVHD) after HSCT, and thus, it is difficult to use these drugs as a bridging therapy to HSCT^28,29^. On the other hands, there are few treatment options for patients without indications for transplantation. These factors suggest that the efficient and safe treatment options for aggressive ATL are not yet sufficient.

We have previously shown that ALA-PDT could selectively kill ATL cell lines established from patients with acute-type ATL and also shown the dynamic changes in flow cytometry profiles during the onset and progression of ATL with patient specimens. Furthermore, 98.7% of ATL leukemic cell death in the chronic ATL patients could be induced with 10 min of visible light exposure, while 77.5% of normal PBMCs survived. Metabolomics analyses revealed that a specific stage of the metabolic pathway progressively deteriorated with HTLV-I infection and at the onset of ATL^30^. Based on the basic findings, we are now in the stage of preparing clinical applications for this treatment. For preparing the clinical trial of ALA-PDT, it is necessary to identify the types and clinical stages of lymphoid malignancies those are the most promising targets for PDT effect. For this reason, in the current study, we obtained blood samples from patients with ATL at various clinical and therapeutic stages, and investigated the effects of PDT on ATL cells. We also evaluated the susceptibility of ATL to ALA-PDT comparing to other hematological malignancies including chronic lymphocytic leukemia (CLL), follicular lymphoma (FL) and Sézary syndrome.

## Results

### PDT efficiently induces necrosis of ATL cells from patient blood

**Figure 1:**
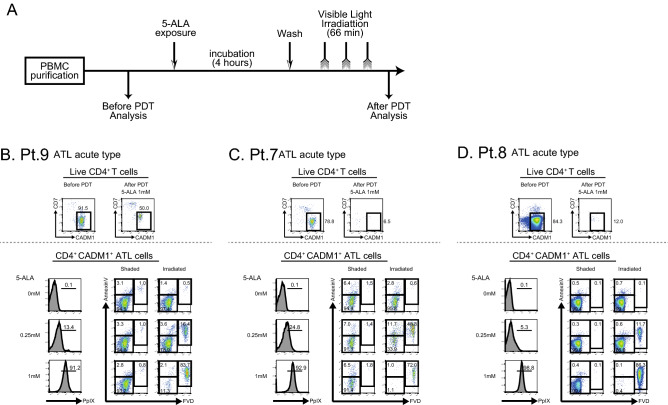
PDT induces necrosis of ATL cells from patient blood. (**A**) The experimental procedure of PDT is shown. PBMCs were purified from peripheral blood samples and then were incubated in the presence of various concentrations of 5-ALA for 4 h. After the removal of 5-ALA, PBMCs were irradiated with 630 nm visible light for 1 h. Samples were promptly analyzed by flow cytometry. (**B**) Analyses of three patients with ATL are shown. Live CD4^+^ T cells show the population of CD4^+^CD7^-^CADM1^-^ cells. ATL cells were identified by CD4, CD7 and CADM1 as shown in the upper panels of Fig. 1B-D. PpIX accumulation on ATL cells after incubation is shown in the lower left panels. Apoptosis and necrosis of tumor cells after PDT are shown in the lower right panels.

To evaluate the effect of ALA-PDT on cancer cells in patients with hematological malignancies, we established an in vitro experimental system (Fig. 1A). In our previous paper, we confirmed that PpIX accumulation in tumor cell lines had reached at 4 h after ALA addition into the culture media, while normal cells did not accumulate PpIX in the same time period^30^. Based on the data, we treated tumor cells for 4 h in the current study. Purified PBMCs were incubated with various concentrations of 5-ALA for 4 h and then exposed to visible light for 1 h. Cell viability was evaluated before and after the ALA-PDT procedure by flow cytometry.

We stained surface CD4, CD7 and CADM1 together with Annexin V and FVD. CD4^+^ T cells were subdivided into 3 subpopulations based on the expression pattern of CD7 and Cell adhesion molecule 1 (CADM1). Previous studies reported that CADM1 is expressed in HTLV-1-infected cells and that the downregulation of CD7 in CADM1-positive cells indicate clonal evolution from an asymptomatic carrier (AC) to ATL^31–33^. Therefore, normal cells, HTLV-1-infected premalignant cells and HTLV-1-infected ATL cells are efficiently enriched in the CD7^+^CADM1^-^ fraction, in the CD7^+^CADM1^+^ fraction and in the CD7^-^CADM1^+^ fraction, respectively. In each population, cells that did not express either Annexin V or FVD were defined as “live cells” in this study. First, CD4^+^Annexin V^-^FVD^-^ live cells were gated and examined for the expression of CD7 and CADM1 (Fig. 1B–D). In the upper panels of Fig. 1B–D, tumor cells in the live cell gate were reduced after the ALA-PDT procedure in all 3 cases. In each case, tumor cells in the live cell gate decreased from 91.5 to 50.0% in patient 9 (Pt.9), from 78.8 to 6.5% in Pt.7, and from 84.3 to 12.0% in Pt.8. In the lower left panels of Fig. 1B–D, the accumulation of PpIX in tumor cells was evaluated. PpIX accumulated according to the concentration of 5-ALA in all 3 ATL acute type cases. In the lower right panels of Fig. 1B–D, the number of necrotic cells did not increase regardless of the concentration of 5-ALA in the shaded state, but after light irradiation, the number of FVD-positive necrotic cells markedly increased according to the concentration of 5-ALA. Of note, the efficacy of PDT was also observed in a patient with prior anti-CCR4 antibody treatment (Fig. S1). These findings suggest that cancer cells from the ATL acute type are very susceptible to necrosis by this ALA-PDT procedure.

### The effect of PDT on indolent lymphoid malignancies varies depending on the levels of tumor cell activation

**Figure 2:**
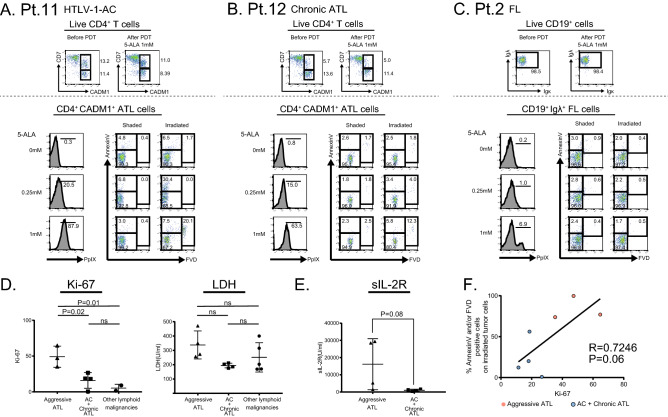
The effect of PDT on indolent lymphoid malignancies was limited in case PpIX accumulation was not sufficient. (**A**)–(**C**) Analyses of three patients with HTLV-1-AC, chronic ATL and FL are shown. Tumor cells were identified as CD4^+^CADM1^+^ cells (**A**), (**B**), and as CD19^+^Igλ^+^ cells in FL (**C**). PpIX accumulation on tumor cells after incubation is shown in the lower left panels. Apoptosis and necrosis of tumor cells after PDT are shown in the lower right panels. (**D**) The percentages of Ki-67 expression on tumor cells (left) and serum LDH levels (right) from patients with aggressive ATL or AC and Chronic ATL or other lymphoid malignancies. (**E**) Serum sIL-2R levels from patients with aggressive ATL or AC and Chronic ATL. (**F**) Correlation between Ki-67 expression in tumor cells before ALA-PDT and % Annexin V and/or FVD positive cells after ALA-PDT (5-ALA 1 mM). Data are expressed as the means +/− SEM.

To evaluate the effect of ALA-PDT on various hematological malignancies other than aggressive ATL, we obtained peripheral blood samples from patients with indolent hematological malignancies as shown in Table 1. In each sample, tumor cell were identified by the tumor-specific surface antigens (Table 1) and evaluated the tumor cell death after PDT. The flowcytometric gating and evaluation strategy in cases of HTLV-1 AC, chronic ATL and follicular lymphoma (FL) are shown in Fig. 2A–C. Our results indicated that the responses of PDT to these tumors were highly variable. For example, in a case of HTLV-1 AC (Pt.11), PDT exposure induced approximately 30% of the CD4^+^CADM1^+^ cells to undergo apoptosis or necrosis (Fig. 2A). In contrast, the percentage of tumor cells in the live cell gate was not reduced after the PDT procedure in Pt.12 and Pt.2 (Fig. 2B,C).

**Table 1:** Patient characteristics.

	Pt. No	Age sex	disease	clinical stage	organ involved	WBC (/μl)	% Ly (PB)	% tumor cells (PB)	LDH	sIL-2R	Prior treatment	Response to prior treatment	tumor cells	%Ki-67^+^ of tumor cells	normal cells	%Ki-67^+^ of normal cells
Other lymphoid malignancies	2	70F	FL	StageIV	BM	2570	74.5	74.5	170	8731	RF RB Bendamustin	PR	CD19^+^IgLammda^+^	–	CD19^+^IgKappa^+^	–
	4	61F	CLL	Rai:II, Bient:A	BM	64,260	91.0	91.0	203	607	Fludarabine Ibrutinib	PR	CD19^+^CD20^+^cells	–	CD19^+^CD20^-^CD23^-^	–
	5	66F	CLL	Rai:I, Bient:B	BM	24,360	83.5	83.5	169	541	RF 6cycle Ofatumumab 12cycle FB 6cycle	SD	CD19^+^CD20^+^cells	1.5	CD19^+^CD20^-^CD23^-^	0.2
	3	77 M	SS	Stage IVA	Skin BM	16,710	14.0	30.5	400	2551	New-Onset	NA	CD3^dim^CD4^–^	–	CD3^+^CD4^+^ CD3^+^CD4^-^	–
	13	74 M	SS	StageIVB	Skin	17,280	6.5	24.0	315	5890	Non-Treated	NA	CD3^+^CD4^+^CCR4^+^	9.0	CD3^+^CD4^+^CCR4^-^	13.3
HTLV-1 AC	10	41 M	HTLV-1 AC	NA	7,400	37.0	0.0	186	315	Non-Treated	NA	CD4^+^CADM-1^+^	18.6	CD4^+^CD7^+^CADM-1^-^	5.4	
	11	39F	HTLV-1 AC	NA	3,930	38.5	3.5	212	659	Non-Treated	NA	CD4^+^CADM-1^+^	18.0	CD4^+^CD7^+^CADM-1^-^	1.3	
Chronic ATL	6	65F	ATL	Chronic	Skin	7430	11.5	3.0	176	1323	Etretinate PUVA NBUVB	SD	CD4^+^CADM-1^+^	26.6	CD4^+^CD7^+^CADM-1^-^	4.5
	12	50F	ATL	Chronic	Skin Breast	1350	87.0	2.0	203	1812	mLSG15 1cycle	NA	CD4^+^CADM-1^+^	0.5	CD4^+^CD7^+^CADM-1^-^	0.3
Aggressive ATL	1	60F	ATL	Acute	BM LN	2610	12.0	0.0	273	1494	MOG 2cycle mLSG15 3cycle	PR	CD4^+^CADM-1^+^	–	CD4^+^CD7^+^CADM-1^-^	–
	9	55 M	ATL	Acute	Skin	30,020	4.3	61.3	471	29,360	Non-Treated	NA	CD4^+^CADM-1^+^	35.3	CD4^+^CD7^+^CADM-1^-^	1.9
	7	39 M	ATL	Acute	BM CNS	7500	6.0	6.3	257	28,481	New-Onset	NA	CD4^+^CADM-1^+^	64.7	CD4^+^CD7^+^CADM-1^-^	2.9
						5340	8.0	1.0	228	3313	VCAP + AMP 1cycle	PR	CD4^+^CADM-1^+^	73.0	CD4^+^CD7^+^CADM-1^-^	4.7
	8	69F	ATL	Acute	Skin BM CNS	38,170	14.0	62.0	350	5264	New-Onset	NA	CD4^+^CADM-1^+^	47.3	CD4^+^CD7^+^CADM-1^-^	6.9
						1900	19.0	21.5	218	1196	mLSG15	PR	CD4^+^CADM-1^+^	18.9	CD4^+^CD7^+^CADM-1^-^	7.1

Percentage of tumor cells were counted on microscopic examination.

*Ly* lymphocytes, *PB* peripheral blood, *FL* follicular lymphoma, *CLL* chronic lymphocytic lymphoma, *SS* sezary syndrome, *AC* asymptomatic carrier, *BM* bone marrow, *CNS* central nervous system, *LN* lymph node, *PUVA* psoralen plus ultraviolet A, *NBUVB* narrow band UVB, *RF* rituximab/fludarabine, *RB* rituximab/bendamustine, *OFA* ofatumumab, *FB* fludarabine/bendamustine, *MOG* mogamulizumab.

We examined the expression of Ki-67 in tumor cells and the serum lactate dehydrogenase (LDH) levels of 13 patients and compared them among the following three groups; aggressive ATL (n = 4), HTLV-1 AC and indolent ATL (n = 4), and other lymphoid malignancies (n = 5) (Fig. 2D). The tumor cells of aggressive ATL were more proliferative than those of other diseases. In ATL patients, the concentration of serum soluble IL-2 receptor (sIL-2R) was relatively higher in patients with aggressive ATL than in patients with indolent ATL patients (Fig. 2E,F). In the analysis of overall patients combined from groups of acute ATL, chronic ATL and HTLV-1 carrier, there was a positive relationship between % Ki-67 and % dead cells after PDT, however, in the analysis of each patient group, there was no correlation between the parameters. (Fig. 2F).

### ALA-PDT eradicates tumor cells but not normal lymphocytes from patients with aggressive ATL

**Figure 3:**
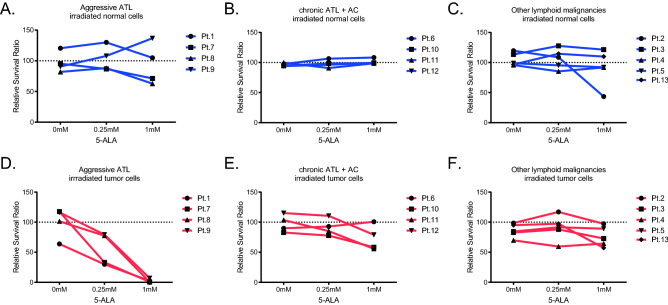
ALA-PDT eradicates tumor cells but not normal lymphocytes from patients with acute ATL. The effects of ALA-PDT on tumor cells and normal cells in the examined 13 patients were summarized. Calculation of relative survival ratio is described in method. (**A**)–(**C**) Relative survival ratio of normal cells in patients was shown in blue. (**D**)–(**F**) Relative survival ratio of tumor cells in patients was shown in red. Relative survival ratio of tumor cells from patients with aggressive ATL was significantly decreased according the concentration of 5-ALA (**D**).

The effects of ALA-PDT on tumor cells and normal cells in the examined 13 patients were summarized in Fig. 3. Treated cells were examined for the expression of Annexin V and FVD, and the components of Annexin V^-^FVD^-^ live cells were calculated. As for aggressive ATL, the percentage of dead cells increased and the percentage of tumor cells decreas ed in the irradiated state with ALA-PDT. The effect was dependent on the concentration of 5-ALA (Fig. 3A). HTLV-1 AC and chronic ATL patient specimen showed the similar dose-dependent decrease of survival leukemic cell percentage after PDT except for one specimen of chronic ATL (Pt.6), which was received skin directed therapies. However, tumor killing activity of PDT treatment was not so strong as that of acute ATL cases. As for other lymphoid malignancies, there were no differences in the components in terms of the amount of 5-ALA or visible light irradiation (Fig. 3B,C).

We calculated the relative survival ratio to compare the effect of ALA-PDT on normal cells and tumor cells under each condition. The definition of normal cells and tumor cells by cell surface markers are shown in Table 1. For other lymphoid malignancies, there were no differences in the relative survival ratios of normal and tumor cells in each condition (Fig. 3F). For aggressive and indolent ATL, the relative survival ratio was the lowest for irradiated tumor cells after incubation with 1 mM 5-ALA. In contrast, the relative survival ratio of normal cells in three disease categories was not influenced by ALA-PDT (Fig. 3A–C), suggesting that ALA-PDT could spare normal cells and selectively kill tumor cells.

### The cytolytic effects of PDT on ATL cells are depending on PpIX accumulation

**Figure 4:**
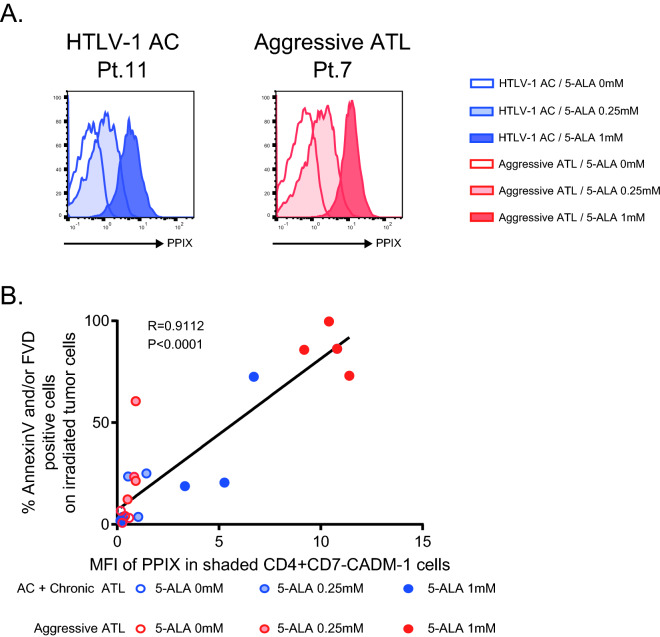
The cytolytic effects of PDT on tumor cells are depending on PpIX accumulation. PpIX accumulation on tumor cells defined by CD4^+^CD7^-^CADM^+^ at each 5-ALA concentration (**A**). Correlation between MFI of PpIX in tumor cells and % Annexin V and/or FVD positive cells in tumor cells after ALA-PDT (**B**).

We evaluated PpIX accumulation in CD4^+^CD7^-^CADM1^+^ ATL cells in each sample by measuring mean fluorescence intensity (MFI). As shown in Fig. 4A, PpIX accumulation increased in an ALA dose-dependent manner both in a HTLV-1 AC and in an acute ATL patient. Importantly, there was a significant linear association between the PpIX accumulation in tumors and the killing effect by PDT, suggesting that the cytolytic effects of PDT on ATL cells are depending on PpIX accumulation (Fig. 4B).

### ALA-PDT is effective for residual ATL cells after conventional therapy

**Figure 5:**
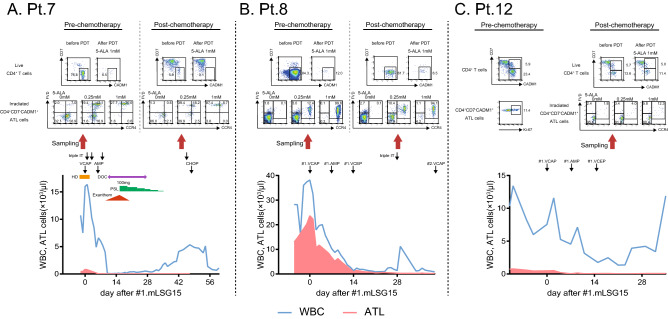
ALA-PDT is effective for residual ATL cells after induction chemotherapy. Clinical course of three patients with ATL were shown. Live CD4^+^ T cells show the population of CD4^+^CD7^-^CADM1^-^ cells. ATL cells were identified by CD4, CD7 and CADM1 as shown in the upper panels. Evaluation of cell death after ALA-PDT are shown in the middle panels. The clinical course is shown in the lower row. *Triple IT* intrathecal injection (methotrexate, cytarabine, prednisolone), *VCAP* vincristine, cyclophosphamide, doxorubicin and prednisolone, *AMP* doxorubicin, ranimustine and prednisolone, *VCEP* vindesine, etoposide, carboplatin, prednisolone, *HD* hemodialysis, *DOC* disturbance of consciousness, *PSL* prednisolone.

In patients with aggressive ATL, the effect of ALA-PDT on chemotherapy-resistant ATL cells that remained after the initial induction chemotherapy was examined (Fig. 5). Two patients with aggressive ATL and one patient with chronic ATL received the standard induction chemotherapy for ATL, which consisted of the following regimens: vincristine, cyclophosphamide, doxorubicin and prednisone (VCAP); doxorubicin, ranimustine and prednisone (AMP); and vindesine, etoposide, carboplatin and prednisone (VECP) (VCAP‐AMP‐VECP; mLSG15)^17^. The clinical course of each patient is shown in the lower panels of Fig. 5A,B. In Pt.7 and Pt.8, 5.9% and 61.7% of the CD4^+^ T cells showed the CD7^-^CADM1^+^ phenotype and were considered to be chemotherapy-resistant ATL populations, respectively, (Fig. 5A,B, upper panels). Our data demonstrated that the number of these cells was markedly reduced after the ALA-PDT procedure. Most of the residual tumor cells after ALA-PDT showed an FVD-expressing necrotic phenotype (Fig. 5A,B, middle panels). Importantly, in Pt.7, the expression of CCR4 in ATL cells decreased after chemotherapy. However, the CCR4-negative residual ATL cells as well as CCR4-positive ATL cells were also sensitive to ALA-PDT (Fig. 5A, middle panels). In contrast to Pt.7 and Pt.8, Pt.12 was clinically diagnosed as indolent ATL with breast involvement, and the proliferative activity of the circulating ATL cells was still low (percentage of Ki-67-positive cells was 11.4%) at diagnosis (Fig. 5C, upper-left panel). In this patient, 13.6% of the CD4^+^ T cells showed the CD7^-^CADM1^+^ phenotype and were considered to be chemotherapy-resistant, but this population was limited to partial reduction after ALA-PDT (13.6–11.4%). In Pt.7 and Pt.8, MFI value showing the degree of PPIX accumulation in tumor cells was high at 11.4 and 10.8, while in Pt.12, it was low at 3.33 (Table 2). These data suggested that susceptibility to ALA-PDT was associated with PpIX accumulation in tumor cells not only before chemotherapy but also after chemotherapy.

**Table 2:** PpIX accumulation and relative survival ratio on normal and tumor cells.

5-ALA (mM)	Pt #	Diagnosis	MFI of PpIX			Relative Survival Ratio (%)														
			Normal cells			Tumor cells			Normal cells						Tumor cells					
									Shaded			Irradiated			Shaded			Irradiated		
			0	0.25	1	0	0.25	1	0	0.25	1	0	0.25	1	0	0.25	1	0	0.25	1
Indolent Lymphoid malignancy	2	FL	0.12	0.22	0.13	0.23	0.32	0.40	100	88.0	43.7	119.6	108.7	43.5	100	113.8	113.0	98.5	117.0	97.4
	4	CLL	0.19	0.19	0.19	0.17	0.17	0.17	100	94.1	99.9	95.8	85.3	91.8	100	94.7	90.4	69.8	59.6	64.5
	5	CLL	0.20	0.20	0.19	0.17	0.17	0.17	100	95.6	87.5	98.9	95.3	92.2	100	95.2	91.9	84.4	91.5	89.2
	3	SS	0.34	0.53	3.02	0.40	0.75	6.15	100	117.8	123.8	113.5	127.7	121.4	100	101.5	103.3	82.8	87.9	72.8
	13	SS	0.26	0.58	2.21	0.23	3.20	12.80	100	105.5	99.9	96.7	114.6	109.9	100	101.9	97.7	95.1	97.4	57.4
HTLV-1 AC	10	AC	0.17	0.34	4.40	0.18	0.54	5.27	100	99.7	104.8	94.5	98.5	98.8	100	106.4	107.0	82.8	77.7	58.3
	11	AC	0.20	1.21	3.97	0.16	1.44	6.71	100	97.0	100.5	99.9	90.9	98.7	100	109.6	98.2	103.6	85.2	55.7
Chronic ATL	6	cATL	0.19	0.24	0.24	0.28	0.29	0.29	100	110.6	108.3	97.6	106.4	108.3	100	92.9	100.9	89.8	92.8	100.7
	12	cATL	0.46	0.74	3.11	0.39	1.04	3.33	100	102.9	104.3	94.1	96.3	99.7	100	95.9	91.0	115.2	110.5	79.1
Aggressive ATL	1	aATL	0.75	1.37	8.38	0.59	0.84	10.40	100	97.7	110.5	120.5	130.0	104.9	100	104.6	141.8	63.9	29.9	2.5
	9	aATL	0.22	1.04	4.84	0.20	0.91	9.19	100	100.3	99.7	90.7	107.9	136.8	100	103.9	106.2	116.3	79.4	6.9
	7	aATL	0.22	1.80	6.20	0.19	0.91	11.40	100	103.1	102.6	95.4	86.8	71.3	100	102.7	105.8	117.5	33.2	0.6
	8	aATL	0.34	0.88	5.29	0.25	0.51	10.80	100	98.7	97.0	82.0	88.2	62.8	100	101.2	105.0	101.5	77.5	0.5

*FL* follicular lymphoma, *CLL* chronic lymphocytic lymphoma, *SS* sezary syndrome, *AC* HTLV-1 asymptomatic carrier, *cATL* chronic ATL, *aATL* acute ATL.

### Light exposure to ATL cells with subcellular localization of 5-ALA induced active caspase-3 generation and mitochondria membrane potential changes

To further examine the biological mechanisms in which ALA-PDT can kill ATL cells, we performed the analyses with laser-scanning confocal microscopes and flow cytometry. First, we incubated TLOm1 cells in the presence of 5-ALA and examine the localization of PpIX in cells (Fig. S2). The result confirmed that PpIX is accumulated inside ATL cells and mainly localized to mitochondria. Then, we exposed 5ALA-treated ATL cell to the light. Flow cytometry analysis indicated mitochondria membrane depolarization (Fig. S3). Laser-scanning confocal microscope (LSM) analysis also demonstrated the depolarization of mitochondria membrane potential at the PpIX accumulated loci and apoptotic body formation in ATL cells after ALA-PDT (Fig. S4). Active caspase-3 was detected by LSM and flow cytometry after ALA-PDT treatments (Fig. S5). The distribution pattern of active caspase-3 was closely localized to that of PpIX. These data visually and quantitatively confirmed the apoptotic and/or necrotic effect of ALA-PDT on ATL cells and supported our findings of the ALA-PDT effects on primary ATL cells from patient blood in the current study.

## Discussion

In this study, we examined the effect of ALA-PDT on ATL cells from freshly obtained peripheral blood. Our data clearly demonstrated that the ALA-PDT procedure exerts selective cytotoxicity for aggressive ATL cells and spares normal lymphocytes. The efficacy of ALA-PDT appeared to be dependent on PpIX accumulation of tumor cells. The results also showed that the ALA-PDT procedure eradicated chemotherapy-resistant tumor cells that remained after induction chemotherapy. These findings suggest the potential of ALA-PDT to act as a novel alternative treatment that can complement chemotherapy and HSCT for patients with aggressive ATL.

Basic and clinical studies on ALA-PDT have been mainly conducted on only solid tumors^31,32^. The effect of ALA-PDT on hematopoietic tumors has been studied mainly using leukemia tumor cell lines. In leukemia tumor lines, 5-ALA induces the accumulation of PpIX, and light irradiation induces cell death^13,14^. It was also reported that different types of hematological malignancies had different responses to ALA-PDT^33^. To explore the clinical application of ALA-PDT for ATL patients, we conducted experiments to investigate the efficacy of this procedure for primary ATL cells as well as other hematological malignancies.

We first examined the samples from patients with aggressive ATL (Fig. 1). The accumulation of PpIX in tumor cells was observed in an ALA dose-dependent manner. After PDT, the number of tumor cells efficiently decreased, and the majority of residual tumor cells showed an FVD-positive necrotic phenotype. The efficacy was similar to our previous experiments that used aggressive ATL cell lines^30^, showing that ALA-PDT is effective for not only established cell lines but also primary ATL cells from patient blood.

We then checked the efficacy of ALA-PDT for tumor cells of various hematological malignancies other than aggressive ATL. We evaluated Ki-67 expression in tumor cells and showed that Ki-67 expression in tumor cells was associated with the treatment efficacy of ALA-PDT, suggesting that highly proliferating tumor cells were more susceptible to death than slowly proliferating tumor cells. Previous studies reported that healthy human lymphocytes had an increased accumulation of PpIX and experience more cell death by light irradiation when activated by PHA or CD3/28 stimulation^34,35^. Our group also reported that the addition of CD3/28 stimulation increases the accumulation of PpIX and cell death of indolent ATL cells^30^. These data suggest that ALA-PDT may require the additional priming procedure to indolent tumor cells for the efficient induction of cell death. %Ki-67 of tumor cells may be useful as a surrogate marker to evaluate the levels of tumor cell priming.

In the current study, HTLV-1 AC and chronic ATL specimen did not show the efficient response against ALA-PDT compared to acute ATL cases. It is apparently contradicting to our previous study, showing that ALA-PDT induced cell death in the most part of ATL cells in the chronic ATL patients, while major part of normal PBMCs survived^30^. There are several possible causes for the discrepancy of the results between our previous and current study. First, the light source was different. Li-Na lamp was used in the previous study, and then we have developed light emitting diode (LED) light source apparatus for the clinical application of PDT. This LED light source was used for the present investigation. The spectrum characters of light source were slightly different between them, although both of them have strong peak of 630 nm. Second, light exposure condition was different. 45.0 mW/cm^2^ light intensity was exposed for 10 min in the previous study, whereas 20.4 mW/cm^2^ light intensity was exposed for 1 h in the present study, Third, time of incubation with 5-ALA was different. The incubation time was 24 h in the previous study, and 4 h in the current study, respectively. Among them, time of incubation with 5ALA seems to be the most important factor which might affect PpIX accumulation. Our previous data suggested that indolent ATL and lymphoma cases showed slower accumulation of PpIX as compared to acute ATL, which may result in the insufficient accumulation in the short incubation time. We therefore consider that the current study does not necessarily indicate that PDT will not work on other tumors. The further optimization of treatment condition in the clinical setting may enable to apply ALA-PDT for indolent hematological diseases as well as aggressive diseases^30^. For clinical application of PDT to hematological malignancies, the careful consideration of algorithms that can efficiently expose 5-ALA to tumor cells in vivo should be required.

To assess the toxicity of 5-ALA itself and ALA-PDT combination therapy on normal lymphoid cells, we examined the viability of normal cells after interventions with 5-ALA and PDT. For indolent lymphoid malignant lymphomas, the viability of both tumor cells and normal cells was not strongly influenced by ALA-PDT (Fig. 3C,F). On the other hand, for ATL, PpIX accumulation and viability of tumor cells decreased in an 5-ALA dose-dependent manner, while PpIX accumulation and viability of normal cells was not influenced (Fig. 3A,B,D,E and Table 2). The viability of tumor cells more decreased in aggressive ATL than in indolent ATL at the same condition. These data showed the tumor-specific cytolytic effects of ALA-PDT especially in aggressive ATL, which is related to that tumor cells accumulated PpIX more than normal cells (Table 2). Of interest, the viability of normal cell was not affected even through normal cells still retain certain levels of PpIX accumulation. It may suggest that there are other underlying mechanisms that can rescue normal lymphocytes from PDT-induced cytotoxicity. However, in the clinical situation, normal lymphocytes are susceptible to the inflammatory environments and the immune activated lymphocyte may accumulate PpIX more than naive lymphocytes. In addition, we found that 5-ALA alone could affect normal cells even without light exposure (Pt.2), indicating 5-ALA itself may have influenced the normal cell survival (Fig. 3C and Table 2). Further mechanistic studies are warranted on the safety of ALA-PDT to confirm that the clinical application of this treatment would not affect normal cells.

We then investigated the importance of PpIX accumulation on the killing effect of ALA-PDT. Our data confirmed that PpIX accumulation is crucial for the PDT-induced tumor cell death. PpIX was more accumulated in acute ATL than in indolent ATL in the present experimental setting. AC, smoldering ATL and chronic ATL have a heterogeneous clinical and biological backgrounds and presumably it explains the heterogeneity of PDT response for indolent ATL. It is again suggested that tumor cell priming and effective 5-ALA exposure are important in chronic ATL.

Last, we examined the effects of ALA-PDT on ATL cells after conventional induction chemotherapy to determine whether chemotherapy affects the effect of ALA-PDT and whether ALA-PDT can work on chemotherapy-resistant cells. A previous study showed that multidrug-resistant leukemia cell lines had no cross-resistance to ALA-PDT^36–38^. Our data demonstrated that the efficacy of ALA-PDT was not affected by previous chemotherapy and that ALA-PDT could eradicate tumor cells that remained after initial chemotherapy. In addition, as we showed in the clinical course of Pt.7 cells (Fig. 5), ALA-PDT worked regardless of the presence or absence of CCR4 expression on tumor cells, suggesting PDT can work without cross-resistance to CCR4-antibody treatment as well as to chemotherapy. These findings strongly support that ALA-PDT can be used together with other conventional therapies and that these therapies may complement each other.

In this study, we showed the direct effect of ALA-PDT on tumor cell death. In clinical settings, the extracorporeal circulation system is required to irradiate circulating tumor cells. Irradiated and dying tumor cells will return to systemic circulation. Many studies have shown that apoptotic tumor cells express the eat-me signal and provoke the immune-based anti-tumor response, including tumor-specific cytotoxic T lymphocytes (CTLs)^39–42^. By using a photopheresis system, ALA-PDT could directly kill tumor cells in peripheral blood, and the necrotic or apoptotic tumor cells returned in patients, which might enhance the systemic anti-tumor immune response. These findings indicate the possibility that PDT may function not only directly on intravascular tumor cells but also indirectly on extravascular tumor cells by invoking anti-tumor immune responses in the host^43,44^. As a step toward clinical application of ALA-PDT to hematological cancer, the current study confirmed the direct-killing effect of ALA-PDT on the primary tumor cells. The future basic and clinical studies need to clarify the immune-related effect by PDT-treated cells to develop this treatment method to the clinical level.

There are several limitations in this study. First, our analysis was performed on a small number of patient samples with limited diseases. The further accumulation of various cases will be important to define the efficient clinical application of this treatment. Second, we made experiments with only one setting for light intensity. Higher light intensity may have been able to induce cell death even in tumor cells with low PpIX accumulation, and inversely, lower light intensity may be enough to induce cell death in tumor cells with high PpIX accumulation. Third, our research did not evaluate cell death other than apoptosis and necrosis. Cell death was defined in many variations^45^. It has been reported that various cell death such as autophagy, necroptosis and ferroptosis were induced by PDT^46–49^. Future studies will need to investigate the details of cell death by ALA-PDT. Fourth, all experiments in this study were performed in vitro. Further studies with different experimental systems might be required to resolve the differences between in vitro and in vivo situations. Finally, the effect of PDT on normal nonlymphoid cells, including neutrophils, erythrocytes, and platelets, has not been well studied. Particularly, erythrocytes will be strongly exposed to PDT in the clinical situation; thus, this effect should be determined in future studies.

In conclusion, these results indicate that PDT using 5-ALA is a unique and distinct therapeutic approach that can be combined with conventional chemotherapy or other emerging therapies, such as anti-CCR4 antibodies, thus providing a novel option to efficiently control the disease status of aggressive ATL.

## Methods

### Patient characteristics

The laboratory studies described in this report were performed for 13 adult patients who were newly diagnosed with lymphoid malignancies at Okayama University Hospital. The patients had circulating cancer cells in the peripheral blood. All patients were enrolled in clinical research protocols approved by the Human Subjects Protection Committee of the Okayama University Hospital. Written informed consent was obtained from each patient prior to sample collection, and this study was in accordance with the Declaration of Helsinki. The clinical characteristics of these patients are summarized in Table 1. One patient had Follicular Lymphoma (FL), 2 patients had Chronic Lymphocytic Lymphoma (CLL), 2 patients had Sezary Syndrome (SS), 2 patients had HTLV-1 Asymptomatic carrier (AC), 2 patients had Chronic Adult T Cell Leukemia (ATL), and 4 patients had Acute ATL. Follicular lymphoma, chronic lymphocytic leukemia and Sézary syndrome were categorized as indolent lymphoid malignancies.

### Experimental conditions for PDT

Peripheral blood mononuclear cells (PBMCs) and nucleated cells (NCCs) were isolated from heparin-treated whole blood samples from the patients. 5-ALA (Sigma Aldrich, St Louis, MO) was diluted in pure water to make a 298-mM stock solution. The cells were incubated in culture medium containing 0, 0.25 and 1 mM 5-ALA at 37 °C for 4 h under light-shielded conditions. After careful wash with 5-ALA-free culture medium (RPMI containing FBS), the PBMCs and NCCs were then exposed to visible light for 66 min for the PDT experiments. The wavelength of the light used was 630 nm, and the light intensity was 20.4 mW/cm^2^; the light was delivered by a plate irradiation device (Otsuka Electronics, Osaka, Japan). The irradiated cells were analyzed by flow cytometry.

### Flow cytometry

Cells in single-cell suspensions were isolated from irradiated samples. The cells were first incubated with directly conjugated monoclonal antibodies (obtained from BioLegend unless otherwise stated) and Fixable Viability Dye eFluor 780 (FVD) (Thermo Fisher Scientific) for 30 min at 4 °C. After 30 min of incubation, the cells were washed twice and incubated with Annexin V PE for 15 min at room temperature. For some experiments, Annexin V labeling was not included.

The following antibodies were used: anti-human CD3 (FITC, clone OKT-3, Thermo Fisher Scientific), CD4 (Pacific Blue, clone PRA-T4), CD7 (FITC, clone CD7-6B7), light chain κ (Pacific Blue, clone 2D1), CD19 (PE, clone HIB19), CD20 (Pacific Blue, clone 2H7), CD23 (FITC, clone EBVCS-5), CD194 (PE-Cy7, clone 1G1,BD Biosciences), light chain λ (FITC, clone MHL-38), and chicken anti-TSLC1/CADM1 antibodies (PE, Clone 3E1,MBL International Corporation, MA, USA).

The cellular PpIX contents were measured at an excitation wavelength of 488 nm and an emission wavelength of 655–730 nm. Cell viability was evaluated with the expression of Annexin V and FVD, each was used as a marker of apoptosis and necrosis, respectively. Cells that did not express either Annexin V or FVD were defined as “live cells”. “The relative survival ratio” was calculated as follows: relative survival ratio = percentage of Annexin V^-^FVD^-^ live cells at each condition/percentage of Annexin V^-^FVD^-^ live cells at 0 mM 5-ALA and light-shielded conditions.

The samples were analyzed on a MACSQuant flow cytometer (Miltenyi Biotec), and the data were analyzed with FlowJo software (Tree Star).

### Statistical analysis

Student’s t-tests were used to assess the statistical significance between 2 groups, one-way analysis of variance was used to assess the statistical significance between 3 groups, and two-way analysis of variance was used to compare more than 3 groups. P values < 0.05 indicated statistical significance. Pearson product-moment correlation coefficient was used for correlation analysis. All tests were two-sided with a significance level of 0.05.

## Supplementary information

Supplementary Information.
