# Author Correction: 5-aminolevulinic acid-mediated photodynamic therapy can target aggressive adult T cell leukemia/lymphoma resistant to conventional chemotherapy

**DOI:** 10.1038/s41598-021-86066-9

**Published:** 2021-03-15

**Authors:** Yasuhisa Sando, Ken-ichi Matsuoka, Yuichi Sumii, Takumi Kondo, Shuntaro Ikegawa, Hiroyuki Sugiura, Makoto Nakamura, Miki Iwamoto, Yusuke Meguri, Noboru Asada, Daisuke Ennishi, Hisakazu Nishimori, Keiko Fujii, Nobuharu Fujii, Atae Utsunomiya, Takashi Oka, Yoshinobu Maeda

**Affiliations:** 1 Department of Hematology and Oncology, Okayama University Graduate School of Medicine, Dentistry and Pharmaceutical Sciences, 2-5-1 Shikata-cho, Kita-ku, Okayama, Okayama 700-8558 Japan; 2 Department of Hematology, Imamura General Hospital, Kagoshima, Japan

Correction to: *Scientific Reports* 10.1038/s41598-020-74174-x, published online 14 October 2020

The Supplementary Information published with this Article contains errors.

The degree of Takashi Oka, “PhD., DMSc”, is incorrectly given as “PhD”.

In addition, the excitation wavelength measurements within the “Supplemental materials and methods” section is omitted.

Lastly, the explanation of the Figures given in the legend of Figures S4 and S5 is incorrect.

The correct Supplementary Information file is linked to this correction notice.

## Supplementary Information

Supplementary Information

