# Supplementary material for: 5-aminolevulinic acid-mediated photodynamic therapy can target aggressive adult T cell leukemia/lymphoma resistant to conventional chemotherapy
Source: Sci Rep. 2020 Oct 14;10:17237. doi: 10.1038/s41598-020-74174-x (PMC7558012; doi:10.1038/s41598-020-74174-x)
Supplement: Supplementary file 1 — Supplementary Information. [file 41598_2020_74174_MOESM1_ESM.pdf]

Supplementary Materials for

5-aminolevulinic acid-mediated photodynamic therapy can target aggressive adult T cell leukemia/lymphoma resistant to conventional chemotherapy

Authors: Yasuhisa Sando[1], Ken-ichi Matsuoka[1*], Yuichi Sumii[1], Takumi Kondo[1], Shuntaro Ikegawa[1], Hiroyuki Sugiura[1], Makoto Nakamura[1], Miki Iwamoto[1], Yusuke Meguri[1], Noboru Asada[1], Daisuke Ennishi[1], Hisakazu Nishimori[1], Keiko Fujii[1], Nobuharu Fujii[1], Atae Utsunomiya[2], Takashi Oka[1*], and Yoshinobu Maeda[1]

Affiliations:
[1] Department of Hematology and Oncology, Okayama University Graduate School of Medicine, Dentistry and Pharmaceutical Sciences, Okayama, Japan
[2] Department of Hematology, Imamura General Hospital, Kagoshima, Japan

* Contributed equally as corresponding authors

This file includes:
Supplemental materials and methods
Supplemental figure legends
Supplemental figures 1-5

Corresponding Authors:
Ken-ichi Matsuoka M.D., Ph.D. and Takashi Oka Ph.D.
Department of Hematology and Oncology, Okayama University Graduate School of Medicine, Dentistry, and Pharmaceutical Sciences, Shikata-cho 2-5-1, Kita-ku, Okayama-city, Okayama, Japan
Phone: +81-86-235-7227; Fax: +81-86-232-8226
E-mail: k-matsu@md.okayama-u.ac.jp (K.M); oka@md.okayama-u.ac.jp (T.O.)

Supplemental materials and methods

Flow cytometry and LSM (laser-scanning confocal microscope) analysis of mitochondria membrane potential changes of TLOm1 cells.

TLOm1 cells, which were established from ATL patient lymphocytes, were maintained in RPMI-1640 medium supplemented with 10% fetal calf serum (FCS; Sankou Junyaku, Chiba, Japan), 100 U/mL of kanamycin (Meiji, Tokyo, Japan), and 100 μg/mL of streptomycin (LIFE Technologies, Rockville, MD, USA). TLOm1 cells were treated with mitochondrial oxidative phosphorylation uncoupler: carbonyl cyanide4-(trifluoromethoxy) phenylhy-drazone（FCCP; Tocris Bioscience, Bristol, UK）0, 10, 20 or 30μM for 30min, 37 ℃ for negative and positive control. For the ALA-PDT analysis, TLOm1 cells were incubated with 1mM 5ALA for 24 h followed by 5 or 10min exposure to LiNa lamp. Then, these cells were treated with JC-1 solution (JC-1 MitoMP detection kit, Dojindo Molecular Technologies, Tokyo, Japan) for 30min, 37 ℃. Mitochondria membrane potential changes induced by FCCP or ALA-PDT treatments were detected with FACSCalibur™ flow cytometer (BD Biosciences, San Jose, CA, USA) and Zeiss laser-scanning confocal microscopes LSM780 (Carl Zeiss Microimaging, Jena, Germany).

Subcellular localization of PpIX in 5ALA-treated TLOm1 cells.

To determine the subcellular localization of PpIX in TLOm1 cells, the mitochondrial dye MitoTracker green FM (Thermo Fisher Scientific, MA, USA) was used. TLOm1 cells were incubated with 1mM 5ALA for 24 h followed by additional culture with 200nM mitochondrial dye MitoTracker green FM for 30min, 37 ℃. Then, cells were washed with PBS and mounted on the slide glass for the observation with Zeiss laser-scanning confocal microscopes LSM780.

Active caspase detection after ALA-PDT treatments with laser-scanning confocal microscope and FCM.

TLOm1 cells were incubated with 1mM 5ALA for 24 h followed by 5 or 10min exposure to LiNa lamp. TLOm1 cells treated with mitochondrial oxidative phosphorylation uncoupler: FCCP, 0 and 30μM, 37 ℃, 30min, were used for negative and positive control. Then, cells were treated with anti-cleaved caspase-3 (Asp175) antibody Alexa Fluor 488 conjugate for 30min, on ice. Cells were washed with PBS and mounted on the slide glass for the observation with Zeiss laser-scanning confocal microscopes LSM780 and also analyzed with FACSCalibur™ flow cytometer.

Supplemental Figures legends

Figure S1. PDT induces necrosis of ATL cells from a patient after receiving mogamulizumab

Analyses of blood sample from Patient 1 with prior mogamulizumab therapy is shown. Live $CD4^+$ T cells show the population of $CD4^+CD7^-CADM1^-$ cells. ATL cells were identified by CD4, CD7 and CADM1 as shown in the upper panels. PpIX accumulation on ATL cells after incubation is shown in the lower left panels. Apoptosis and necrosis of tumor cells after PDT are shown in the lower right panels.

Figure S2. Subcellular localization of PpIX in 5ALA-treated TLOm1 cells.

TLOm1 cells were incubated with 1mM 5ALA for 24 h followed by additional culture with 200nM mitochondrial dye MitoTracker green FM for 30min, 37 ℃. Then, cells were washed with PBS and mounted on the slide glass for the observation with Zeiss laser-scanning confocal microscopes LSM780.
a): MitoTracker green FM signals indicating mitochondria localization. b): PpIX signal, c): Differential interference contrast microscope; (DIC), d); marge, yellow signals indicate co-localization of MitoTracker green FM signals and red PpIX fluorescence. Bars indicate 10μm.

Figure S3. Flow cytometry analysis of mitochondria membrane potential changes of TLOm1 cells after ALA-PDT.

TLOm1 cells were treated with mitochondrial oxidative phosphorylation *uncoupler:* FCCP a): 0μM, b): 10μM, c): 20μM, d): 30μM, 37 ℃, 30min, for negative and positive control. As the ALA-PDT analysis, TLOm1 cells were incubated with 1mM 5ALA for 24 h followed by e): 0min, f): 5min, g): 10min exposure to LiNa lamp. Then, these cells were treated with JC-1 solution for 30min, 37 ℃. Mitochondria membrane potential changes induced by FCCP or ALA-PDT treatments were detected with FCM. Both FCCP and ALA-PDT treatments increased FL1 green fluorescence, indicating mitochondria membrane depolarization.

Figure S4. Laser-scanning confocal microscope (LSM) analysis of mitochondria membrane potential changes of TLOm1 cells after ALA-PDT.

TLOm1 cells were treated with mitochondrial oxidative phosphorylation *uncoupler:* FCCP A): 0μM, B): 30μM, 37 ℃, 30min, for negative and positive control. As the ALA-PDT analysis, TLOm1 cells were incubated with 1mM 5ALA for 24 h followed by 10min exposure to LiNa lamp. Then, these cells were treated with JC-1 solution for

30min, 37 ℃. Zeiss laser-scanning confocal microscopes LSM780 was used for the detection of subcellular localization of JC-1 and PpIX signals. a); 492-560nm: green for JC-1 mitochondria membrane depolarization signal, b); 561-605nm: orange for JC-1 signal, c); 560-610nm: red for PpIX, d); Differential interference contrast microscope; (DIC), e); marge, yellow signals indicate co-localization of green JC-1 signal and red PpIX fluorescence. Bars indicate 10μm.

Figure S5. Active caspase-3 detection after ALA-PDT treatments with LSM780 and FCM.

TLOm1 cells were treated with mitochondrial oxidative phosphorylation uncoupler: FCCP A): 0μM, B): 30μM, 37 ℃, 30min, for negative and positive control. For the ALA-PDT analysis, TLOm1 cells were incubated with 1mM 5ALA for 24 h followed by 10min exposure to LiNa lamp. Then, cells were treated with anti-cleaved caspase-3 (Asp175) antibody Alexa Fluor 488 conjugate. Cells were analyzed with Zeiss laser-scanning confocal microscopes LSM780. a); 492-560nm: green for anti-cleaved caspase-3 (Asp175) Alexa Fluor 488, b); 560-610nm: red for PpIX, c); Differential interference contrast microscope; (DIC), d); marge, yellow signals indicate co-localization of green: anti-cleaved caspase-3 and red: PpIX fluorescence. Bars indicate 10μm. E, FCM analysis of anti-cleaved caspase-3 (Asp175) antibody Alexa Fluor 488 with light exposure 0, 5 or 10min to 5ALA- treated cells.

Figure S1

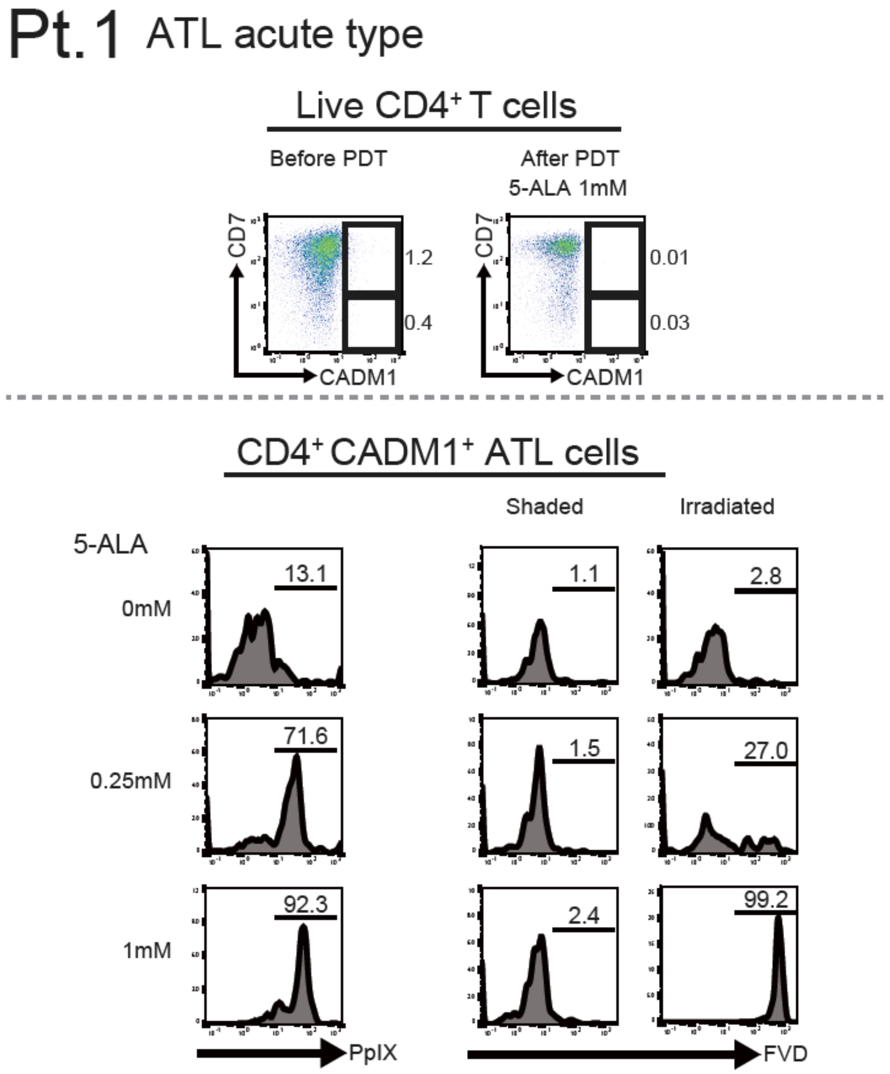

Pt.1 ATL acute type
Live CD4+ T cells
Before PDT
After PDT
5-ALA 1mM
CD7
CADM1
1.2
0.4
0.01
0.03
CD4+ CADM1+ ATL cells
Shaded
Irradiated
5-ALA
0mM
0.25mM
1mM
13.1
1.1
2.8
71.6
1.5
27.0
92.3
2.4
99.2
PpIX
FVD

Figure S2

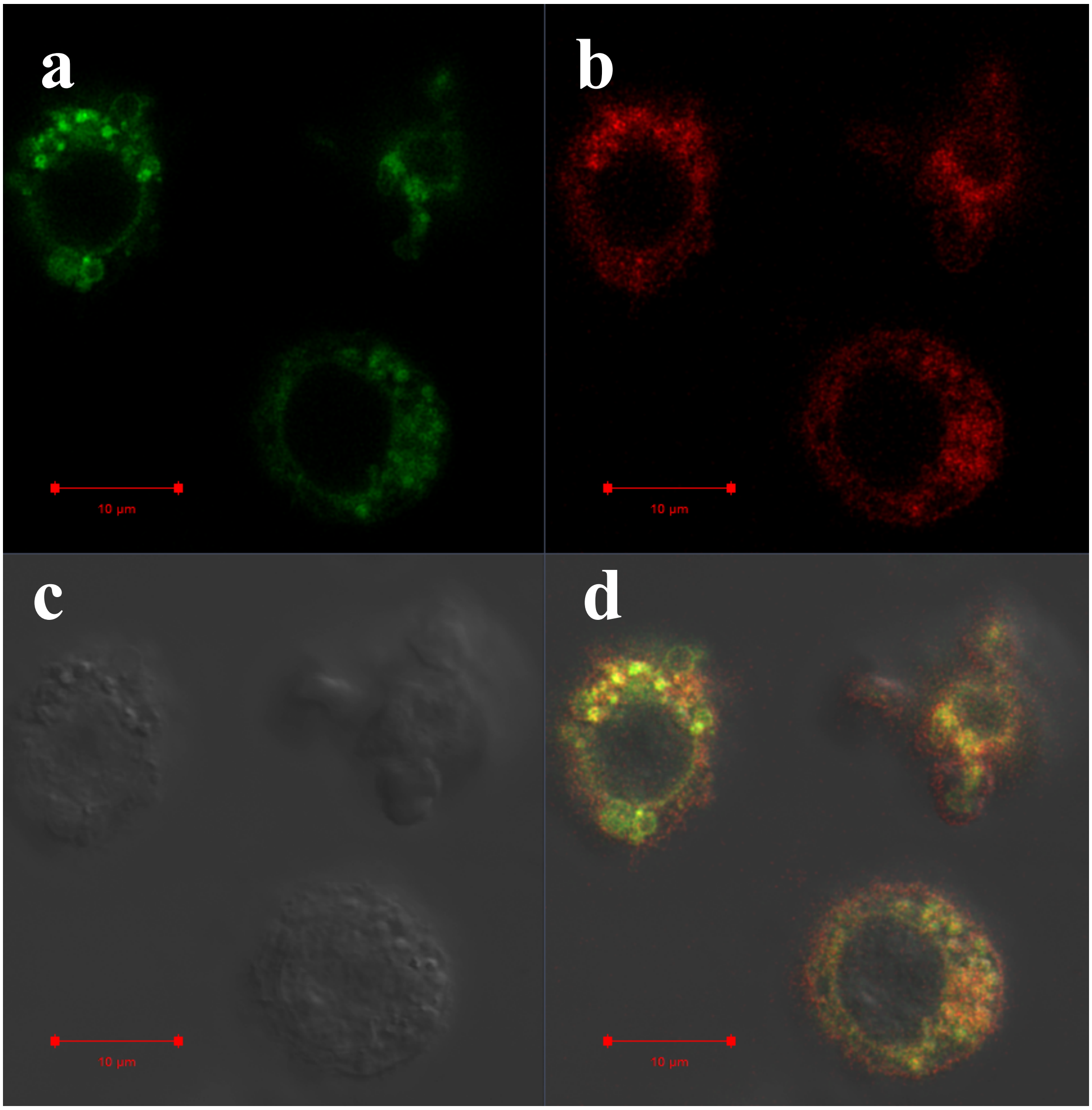

Figure S3

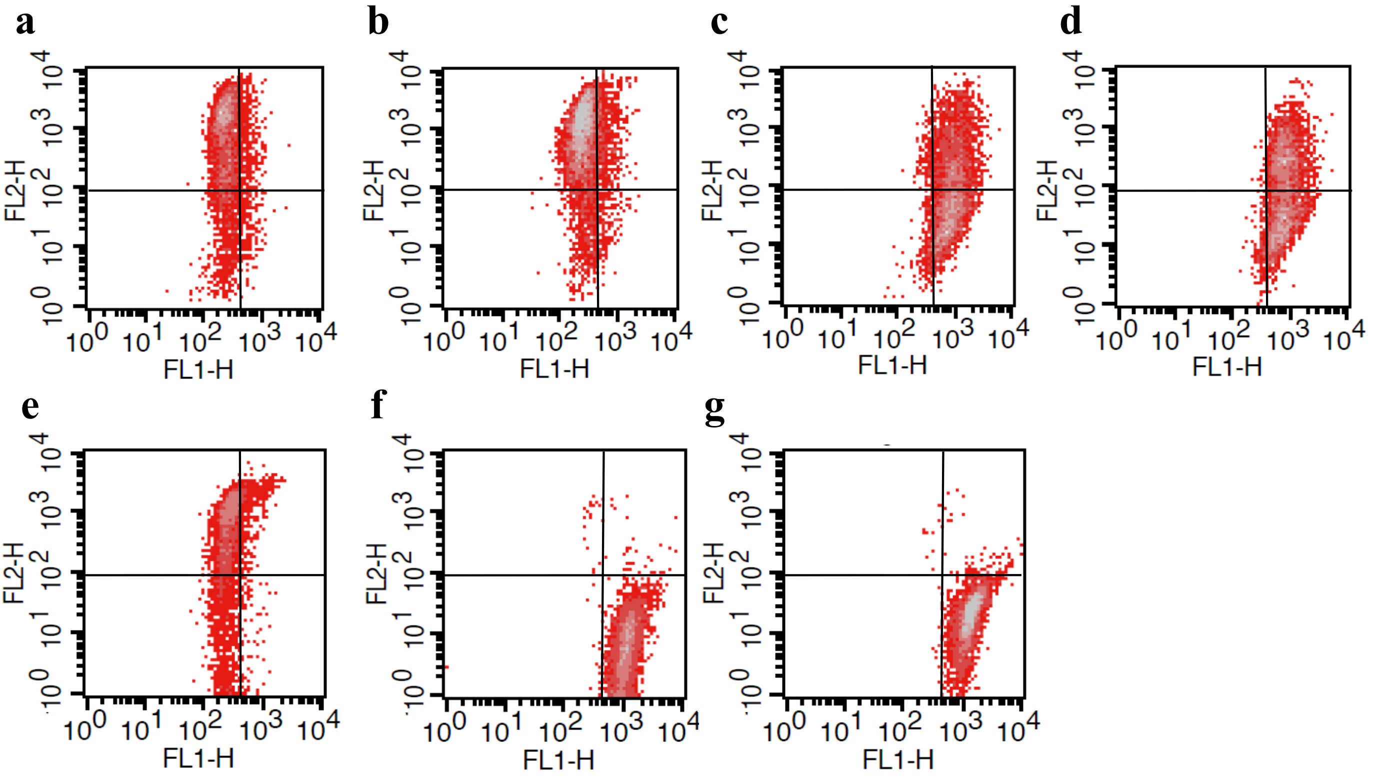

Figure S4

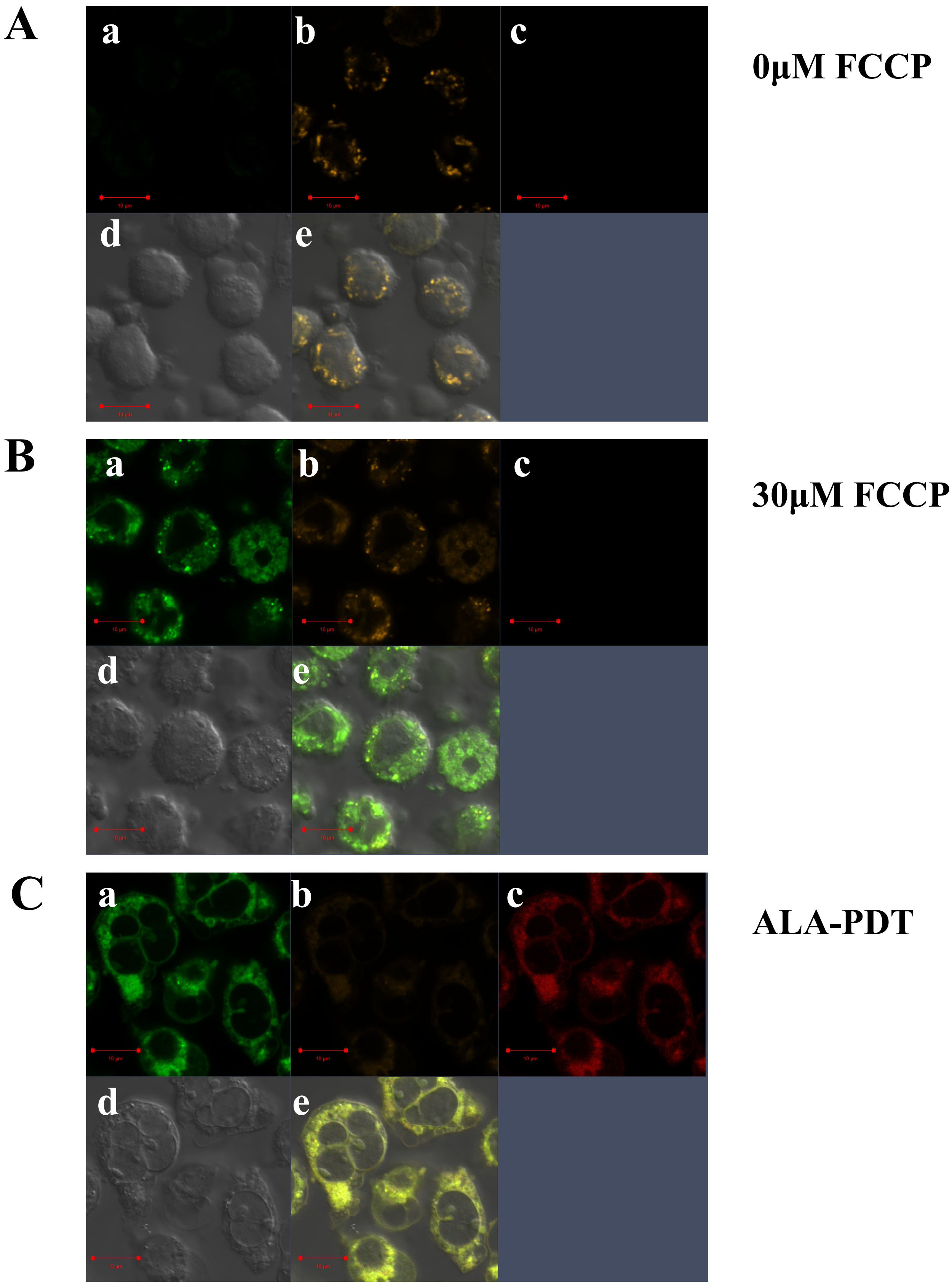

Figure S5

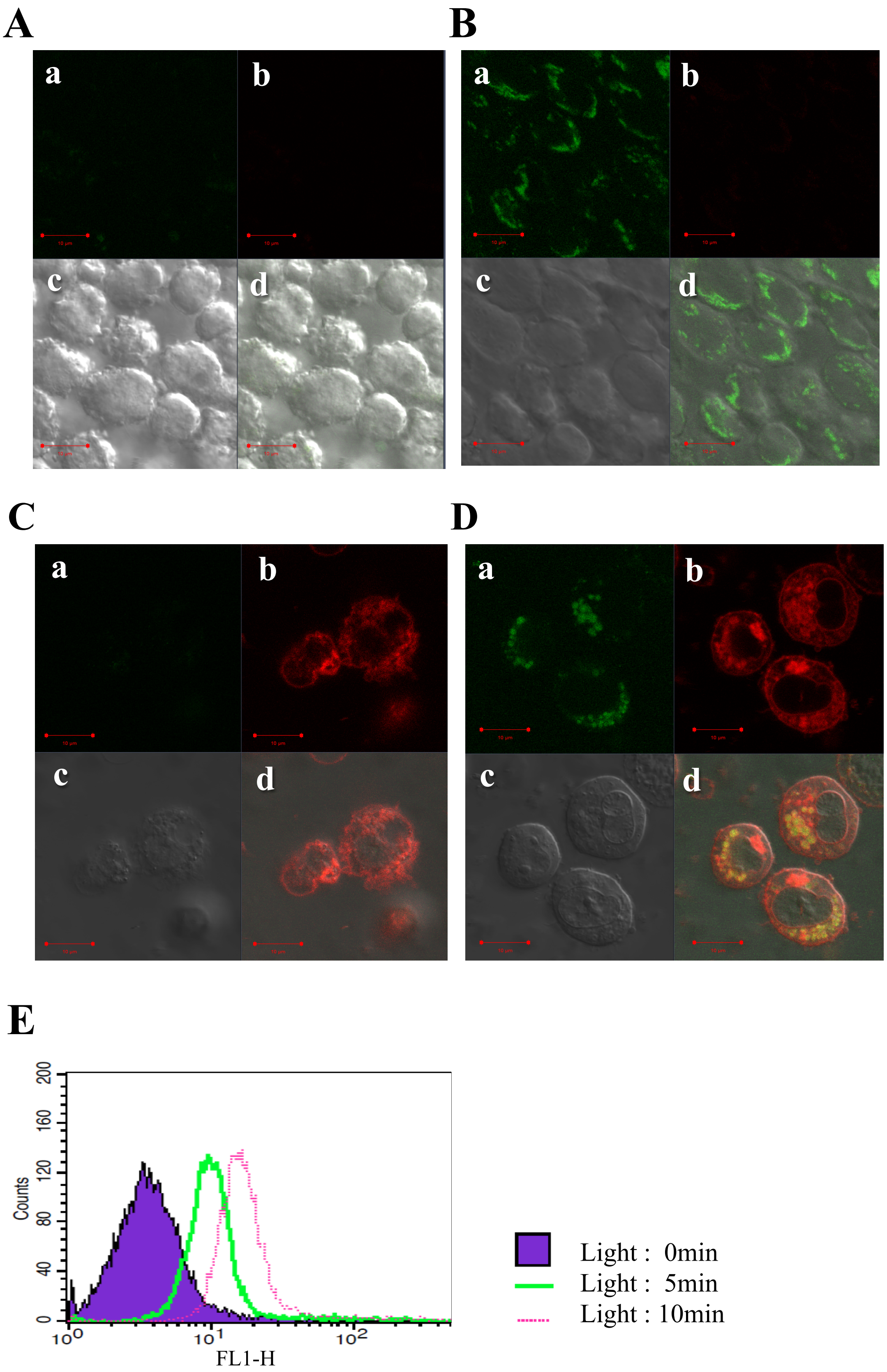
A
B
C
D
E
a
b
c
d
Counts
0
40
80
120
160
200
10^0
10^1
10^2
FL1-H
Light : 0min
Light : 5min
Light : 10min
